# Barriers to linking high-risk jail detainees to HIV pre-exposure prophylaxis

**DOI:** 10.1371/journal.pone.0231951

**Published:** 2020-04-17

**Authors:** Nickolas D. Zaller, Taylor L. Neher, Makenzie Presley, Heather Horton, S. Alexandra Marshall, Melissa J. Zielinski, Lauren Brinkley-Rubinstein

**Affiliations:** 1 Department of Health Behavior and Health Education, University of Arkansas for Medical Sciences, Little Rock, Arkansas, United States of America; 2 Psychiatric Research Institute, University of Arkansas for Medical Sciences, Little Rock, Arkansas, United States of America; 3 Department of Social Medicine, University of North Carolina, Chapel Hill, North Carolina, United States of America; 4 Center for Health Equity Research, University of North Carolina, Chapel Hill, North Carolina, United States of America; Beth Israel Deaconess Medical Center/Harvard Medical School, UNITED STATES

## Abstract

Individuals involved in the criminal justice (CJ) system continue to be at disproportionate risk for HIV infection, and often have a greater prevalence of substance use and sexual related risk behaviors relative to their non-CJ involved peers. Pre-exposure prophylaxis (PrEP), a once daily antiretroviral medicine, is an evidence-based approach for reducing the risk of contracting HIV but limited data exist regarding the use of PrEP among CJ populations, especially in the U.S. South. This study was conducted at the Pulaski County Regional Detention Facility (PCRDF) in Little Rock, Arkansas (AR), the largest county jail in the state. We explored knowledge about PrEP and HIV, perceptions about PrEP feasibility in both the jail and community settings and barriers to PrEP program implementation, through in-depth qualitative interviews with 21 jail detainees. We purposively sampled individuals based on specific self-reported risk behavior, including sexual risk (both heterosexual and same-sex) and drug related risk (e.g. IDU), among all eligible individuals. We identified five primary themes from the interviews: 1) accessing healthcare during community reentry was a low priority; 2) perception of risk and interaction with people with HIV was low; 3) there are many barriers to disclosing HIV risk behaviors in jail settings; 4) knowledge of PrEP is low but willingness to use is high; and 5) multiple barriers exist to PrEP uptake post-release. Our findings are contextually unique and therefore have important implications for future implementation of PrEP access either within jail settings or linkage to PrEP post release.

## Background

Human Immunodeficiency Virus (HIV) continues to be a significant public health issue in the United States (US), especially among criminal justice (CJ) populations. People who are incarcerated have a prevalence of HIV that is 2.4 times that of the general population [1] and post-release is known to be a time of increased engagement in high-risk HIV behaviors (e.g. return to drug use and condomless sex) [2, 3, 4, 5, 6]. For example, one study found that previously incarcerated men had a higher frequency of condomless sex and sex while under the influence of drugs or alcohol relative to men who had never been incarcerated [7]. Recent studies among people who were released from incarceration within the past 12 months have also documented high prevalence of having multiple partners, engaging in condomless sex, injection drug use and having partners who also injected drugs [8].

In the US South, risk of post-release HIV acquisition may be even higher given that this region has some of the highest rates of both HIV and incarceration in the nation. All of the top seven states with the highest incarceration rates are in the South, as defined by the US Census (Louisiana, Mississippi, Georgia, Alabama, Texas, Oklahoma and Arkansas) [9]. Additionally, states in the southern US accounted for 52% of new HIV infections in 2017 [10]. Racial disparities in both HIV and incarceration are also heightened in the South. For example, in the South, more than half of new HIV diagnosis are among individuals of color [10]. Furthermore, African Americans represent 42% of the prison/jail population despite representing 15% of the population in Arkansas, similar to other southern states [11].

Pre-exposure prophylaxis (PrEP), a once daily antiretroviral medicine, is an evidence-based approach for reducing the risk of contracting HIV. When taken consistently by HIV-negative individuals it can reduce the risk of HIV acquisition by 92% [12]. When adherence is low, PrEP is less efficient [12]; for example, if PrEP is taken twice per week, it only reduces risk by 76% [13]. PrEP access also continues to be an additional barrier, especially in the South where access to and uptake of PrEP has been limited [14]. A recent study showed there are fewer than 12 PrEP users per 100,000 population in Arkansas. This translates to a low PrEP-to-Need ratio of ~0.5–1.0 (PrEP users per New HIV Diagnoses) in Arkansas, which is approximately four times less than in the Northeast and the West [14].

Despite its potential benefit, linkage to and use of PrEP among people who are CJ-involved has been limited and there have been numerous barriers to implementation. For example, knowledge about PrEP among those who are incarcerated remains low [6, 15]. Other PrEP related barriers, including medication costs, stigma associated with PrEP, competing needs upon community reentry, concern for side effects and low perceived HIV risk, have also been documented [6; 16–18].

Importantly, studies have documented willingness to take PrEP among individuals who are or become aware that it can prevent HIV infection. A study of incarcerated MSM (men who have sex with men), gay and bisexual men found there was marked interest in using PrEP despite limited familiarity with PrEP [6]. Therefore, the goal of our study was to advance knowledge regarding the facilitators and barriers to implementing a PrEP linkage intervention specifically upon reentry from a county jail in the South. We explored knowledge about PrEP and HIV, perceptions about PrEP and barriers to PrEP program implementation, through in-depth qualitative interviews.

## Methods

This study was conducted at the Pulaski County Regional Detention Facility (PCRDF) in Little Rock, Arkansas (AR). The PCRDF is Arkansas’ largest county jail and processes about 25,000 detainee intakes each year. At the time of this study, the PCRDF did not offer HIV testing or any HIV prevention related programming within the facility or linkage to such programming upon release to the community.

### Recruitment and enrollment

Between January and June 2018, we worked with PCRDF intake staff to initiate a process to screen detainees at intake for their HIV risk according to the Centers for Disease Control and Prevention (CDC) practical guidelines for PrEP [12]. Prior to initiating screening, PRCDF staff (including all correctional officers), were informed about the study aims and methods and were asked to refer potentially eligible detainees (if individuals endorsed HIV risk during the screening process) to a member of the study team during times when study team members were present at the PRCDF, which was typically in the morning three to four days per week.

### Description of the HIV risk (PrEP eligiblity) screening process

The HIV risk screening took place while individuals were being processed into custody by intake personal who also conducted medical screenings (i.e., took a brief health history including medications, screened for behavioral health disorders). The HIV screening assessment contained 10 questions about behavior in the 6 months prior to the current incarceration. The screening, based on published CDC guidance [12] was administered either orally or through pen and paper, based on the individual’s preference. Screening questions included self-reported measures of race, gender, HIV status, last HIV test, perceived risk for HIV, sexuality and sexual behaviors, STI history, drug use (including history of IDU), and PrEP knowledge. The screening was intended for all individuals admitted to the jail during their medical screening (which usually occurs between 24–72 hours after jail intake). The medical screenings conducted by the jail are performed by trained (including HIPAA trained) medical personnel to gather information on the individual’s current medical needs. In addition, the medical personnel are contracted through a third party vendor and are not employed by the jail. However, since the HIV screening was not a mandatory procedure, medical personnel may have selectively administered the HIV risk screening questionnaire, based on time constraints, due to the high volume of people booked in to our study site each day. No additional resources, information, or testing (including HIV testing) was provided upon completion of the screening; the HIV screening was outside the scope of what the medical staff are contracted to do and as such, additional efforts to engage individuals around their HIV risk would have meant a significant increased burden of work on the part of medical personnel, which has costs and other logistical implications. The jail does not provide routine HIV testing, if an individual requests an HIV test, the Arkansas Department of Health is contacted and arranges for testing within the jail.

There were approximately 14,572 individual intakes and 3,069 HIV risk screenings conducted during the study period (21.6% of the total number of intakes). People who were determined to be eligible for PrEP based on the screening were identified by study staff from the screening and were asked by the PRCDF correctional guards about their interest in participating in a health and wellness research study consisting of a one-time interview. The choice to describe the study as a health and wellness study was made in order to avoid the potential stigma associated with wording related to HIV or PrEP (and to maximize safety and confidentiality of study participants). Interested individuals were then brought to a private area, where no correctional officers were present, within the medical unit of the PCRDF to discuss the study in more depth with a member of the research team and to confirm self-reported HIV risk behaviors. Inclusion criteria for interviewees included: being age 18 or older, having the ability to understand English, and being able to give informed consent. Additionally, we purposively sampled individuals based on specific self-reported risk behavior, including sexual risk (both heterosexual and same-sex) and drug related risk (e.g. IDU), among all eligible individuals. Purposive sampling was used to ensure the sample was diverse with respect to HIV risk and gender. We sought to interview 12 males divided equally between African American and white individuals with 4 from each HIV risk category we included within our study eligibility criteria: MSM, heterosexual risk, and IDU. Sampling frames were the same for females with eight individuals divided equally between African American and white females with 4 from each risk category of heterosexual risk and IDU. Once the eligibility determination was made and specific risk behaviors identified, participants were given more information about the study and consented. Of the 3069 individuals screened, 91 met the inclusion criteria and reported one or more of the HIV risk factors we specified in our sampling frame. Given the transient nature of the jail, 66 individuals were released from custody prior to us having the opportunity to interview them, thus making them ineligible for the study. Five people refused to participate in the study and three people were unable to participate due to various reasons including the location they were incarcerated in the jail or insufficient numbers of guards available for transport on days in which we were conducting interviews. Our final sample consisted of 21 detainees.

### Data collection

Data collection consisted of a one-time in-depth interview using a semi-structured interview guide. Interviews lasted on average 45 minutes and were conducted by members of the study team trained in qualitative methods (NZ and TN). Interview domains included priorities post-release from incarceration, healthcare usage and access, HIV knowledge and awareness, PrEP knowledge and interest, barriers and facilitators to taking PrEP while incarcerated and/or in the community, and attitudes about a potential intervention utilizing community health workers (CHWs) to link detainees to PrEP post-release (CHW results to be presented in a separate manuscript). All interviews were digitally recorded and transcribed verbatim. Participants were credited $30 in their commissary accounts at the PCRDF as an incentive. The University of Arkansas for Medical Sciences institutional review board approved this study before it commenced.

### Analysis

Data were analyzed in MAXQDA using a thematic analysis, a qualitative method in which themes from both the research questions and the narratives of the research participants are generated [19, 20].The preliminary codebook was refined after the first three transcripts were completed using methods developed by Carey, Morgan, and Oxtoby [21]. Coding of the interviews was informed utilizing a general inductive approach that allowed for themes and categories to be identified. Coders (MZ, LBR, NZ, SAM, and TN) analyzed the transcribed data of participants that had similar HIV risk factors (e.g. injection drug use) to determine reoccurring themes and patterns. This process was utilized to establish themes and sub-themes with attached codes to develop the codebook for the remainder of the transcripts. All transcripts were coded in an iterative process by two researchers coding separately and identifying discrepancies (NZ and TN). In addition, 20% of the transcriptions had quality checks conducted with a third team member (MZ). Discrepancies within the initial set of interviews were resolved by consensus via meetings with the full study team to finalize the codebook. After all coding was completed, codes were organized into themes which were subsequently analyzed until thematic saturation was achieved.

## Results

Our total sample consisted of 21 detainees with an average age of 35.6 years (range 21–58). Nine participants (43%) were African American and 13 (62%) were male (Table 1). With respect to HIV risk, nearly half reported heterosexual risk factors of having unprotected sex with multiple partners of a different gender (N = 10, 48%) and 9 participants (43%) reported IDU behavior. MSM participants were underrepresented with only three (23% of male participants) identifying as MSM (Table 1). Thematic differences were not identified based on specific HIV risk groups however, we provide risk factors of the participants after each representative quote provided below to provide brief description of individual risk profiles.

**Table 1:** Participant demographics.

		Males (n = 13)	Females (n = 8)	Total % (n = 21)
**Average Age**	(range 21–58)	38.08	31.63	35.62
**Race**	White	5	6	52%
	Black/African American	7	2	43%
	More than 1 Race	1	0	5%
**Hispanic/Latino**	Yes	0	1	5%
	No	13	7	95%
**MSM (males only)**	African American	2	n/a	23% of males
	White	1	n/a	
**IDU**		5	4	43%
**Sexual Risk Behaviors**		5	5	48%
**STI diagnosis in the last 6 months**		4	2	29%

We identified five primary themes from the interviews: 1) accessing healthcare during community reentry was a low priority; 2) perception of risk and interaction with people with HIV was low; 3) there are many barriers to disclosing HIV risk behaviors in jail settings; 4) knowledge of PrEP is low but willingness to use is high; and 5) multiple barriers exist to PrEP uptake post-release.

### Accessing healthcare during community reentry was a low priority

Participants discussed competing needs, including housing, employment, reuniting with family and/or significant others as their top concerns during community reentry. Few explicitly discussed healthcare as a priority during community re-entry. Among those who did report having a chronic medical condition requiring ongoing medical care, most described their medical care in the period prior to their incarceration as intermittent or sporadic largely due to inconsistent insurance status. For example, one participant with bipolar disorder commented:

*For me*, *my battle*, *too*, *was a lot with bipolar*. *I wouldn’t stay on my medication*. *If I’m manic*, *then it would be—I’d be gone*, *and three days later*, *and not even know where I was and be high*. *It’s just a roller coaster*. *There’s no doctor*. *There’s no insurance*. *How do you get it*? *How do you stay on your medication to keep you safe and keep you healthy*? *43 year old White Female- Sexual Risk*

For most participants, immediate priorities during community reentry, such as employment and housing, were more important than accessing healthcare. One participant reported,

*I am homeless*, *and I didn’t have a job for nine years*. *It’s been hard to find a job since I quit that job*. *Yes*, *definitely a job*. *Definitely housing*. *Car*. *Get back in society like a normal person*. *30 year old White Female- IDU*

Employment in particular was a key priority most participants discussed at length during their interviews. For example, one participant noted:

…*once you get outa jail you’re steady tryin’ to get yourself back to bein’ on track again*, *as far as work*, *‘cause it’s pretty sure*, *if you did have a job*, *you done lost it sittin’ in here…so now you’re out on the grind of tryin’ to find another job*. *You might have lost your place to live*, *your apartment*, *and now you gotta find another apartment or whatever*. *40 year old More than one race Male- IDU*

In addition, participants commented about how other aspects of their lives, such as continued drug use, interfered with their ability to prioritize health. One participant stated:

*The thing that messes me up with that*, *though*, *is the street drugs*. *I was supposed to be takin’ drugs to help me with a lotta different things I had goin’ on with me*. *Unfortunately*, *I would screw all that up with narcotics and stuff like that that I’d get messed up on*. *It just cancels out everything good in my life*. *50-year-old White Male- IDU*

### Perception of risk and interaction with people with HIV was low

Few participants reported that they knew someone with HIV when asked, and few indicated that HIV had impacted them personally. The overall perception of personal HIV risk was variable; many participants indicated that they were either not at risk or no longer at risk because they were not currently engaging in risky behaviors. One participant put this within the context of injection drug use. He said:

*Because I don’t share needles*. *I don’t do that*. *I’m not gonna—just downtown junkie…*.*I use one needle*, *throw it away*. *47-year-old White Male- IDU*

Another participant shared the same notion of not being at risk even with their prior MSM behaviors but worries about others:

*I have not had interactions with anyone that I knew that had HIV*. *I’m not too worried about it*. *I’m not sexually promiscuous*. *I don’t share needles*. *I’m very safe when it comes to this stuff…*. *I do have some friends that I do worry about*. *That is their choice*, *their decisions*. *I try to be safe and try to lead by example*, *and hopefully*, *they can pick that up and—yeah*. *32-year-old White Male–MSM*

Other participants felt that they were not ‘members’ of the communities most at risk for HIV. For example, one participant did not feel as though she was at risk for HIV infection because she was not gay or an IDU, both risk categories she identified as being most associated with HIV. She commented:

*HIV is really not exposures to the normal*, *everyday working class*. *It’s pretty big with IV [intravenous drug] users and the gay community and prison really*. *You’re startin’ good right here to be honest*. *If you ever wanted to progress*, *honestly*, *I think the next thing if y’all to jump into the gay community*. *That’s just bein’ honest*. *32-year-old African American Female- Sexual Risk*

This comment suggests this participant associated HIV risk and HIV infection with more stigmatized groups such as IDU and MSM populations. Still other participants stated that they hadn’t really thought about their own HIV risk. One participant stated:

*I never considered it*, *honestly*. *I never considered to get HIV*. *Even though I’ve had prior STDs*, *it’s something I never actually thought about*. *20-year-old White Female- Sexual Risk*

### Barriers to disclosure of HIV risk

While participants indicated that many people in jail engaged in high-risk behaviors either prior to being incarcerated or while incarcerated, there are barriers to disclosing specific risk behaviors. Some participants felt comfortable disclosing their own risk behaviors, mostly pertaining to drug use. Others commented that stigma prevents many from disclosing specific risk behaviors in the jail setting. With respect to drug use, some participants did not perceive any barriers to disclosing their prior drug use, perhaps because they were incarcerated for drug related charges or because they felt that most people already knew that they used drugs. As one participant remarked: “I am a drug addict. Why am I gonna lie about it?” (47-year-old *White Male- IDU*).

However, other participants indicated significant stigma associated with HIV risk behaviors. One participant not only felt stigma associated with HIV risk, but also protective of her privacy. She stated:

*I think it would be hard [to disclose] just because I’m so ashamed of myself*. *I’m so embarrassed to tell people that I’ve slept with multiple men within probably a year and a half*. *I feel like it wouldn’t be anybody’s business as well*. *20-year-old White Female- Sexual Risk*

Another participant expressed concerns about their privacy and stigma associated with their behaviors while incarcerated. He stated:

*I don’t ever let my business out like that*. *I was never—I was sexually active with a man once when I was prison*, *the very first time*, *but that was it*. *It’s because I was locked up*, *but that’s irresponsible*. *I was locked up so long that I was making it okay in my head to do that*, *if that makes sense to you*, *but it wasn’t okay for me to do that*, *not from a godly standpoint*. *44-year-old White Male -MSM*

Other participants articulated stigma associated with specific types of behaviors, which in turn led to shame. One participant summarized this when talking about her peers who were IDUs by stating:

*A lot of people is gonna feel ashamed of saying*, *“Well*, *I’m a junkie*. *I shoot*. *I shoot my dope up with the IV*. *That’s the only way I use it*.*” I’ve run into a lot of females that’s in my cell that don’t do nothing but use needles—heroin*, *meth*. *35-year-old White Female- Sexual Risk*

### Knowledge of PrEP was low but willingness to take PrEP was high

Few participants had heard of and/or could accurately describe PrEP. One participant said: *…unless I'm really*, *really way off*, *I don't know that a lot of people in jail*, *maybe because of the lifestyle or circles that we're in*, *don't really have any awareness of that*. *57-year-old African American Male- Sexual Risks*

However, after being told about PrEP, most participants expressed an interest in using PrEP both prior to and post-release. A participant said:

*Well*, *I would hope I wouldn’t have to take it forever*. *I guess that would be something I would have to learn*. *How long do you take it for*? *If you stopped doing—well*, *I guess we’re always gonna—I’m always gonna do suspicious activity*. *I’m not married and I have sex*. *30-year-old White Female- IDU*

Some, though, misunderstood the indications for PrEP stating that they would prefer to use PrEP only during times when they perceived themselves to be at risk for HIV in the community. A participant commented:

*PrEP is a pill I wouldn't take every day*. *PrEP is a pill that I would take like twice out of a week and that would be mostly on the weekends*. *20-year-old African American Male- Sexual Risk*

Many participants preferred to initiate PrEP prior to release and thought this might help to overcome any potential barriers to PrEP during community re-entry.

*I’d probably wanna start it in the jail*. *If you have the opportunity to start it*, *why not take advantage of what they’re actually offering in here*? *Cause if you go out*, *you don’t have the resources to get that medication* … *20-year-old White Female- Sexual Risk*

### Potential barriers to PrEP access and use post-release

After people were asked about their knowledge of PrEP and willingness to learn more, the interviewers provided a brief overview of the PrEP medication including the process of getting a prescription, medication costs, and other potential barriers such as health insurance and transportation. The most common barriers cited by participants regarding PrEP access included: insurance or cost, potential side effects of the medication, and worries about medication adherence. Another concern endorsed by many participants was the need to take PrEP everyday, especially within the context that their perceived HIV risk may vary over time. One participant summarized this as follows:

*Yeah*, *it’s gonna be very hard to get some people to just remember to take that drug throughout the lifespan of how long they’re trying to take it because if they’re not being exposed or if they’re not*, *if they don’t have HIV*, *they’re not gonna remember really because they’re*, *“Well*, *I didn’t do anything today*. *I didn’t expose myself*, *so I didn’t have to take it*.*” 32-year-old African American Female- Sexual Risk*

Another participant commented that her addiction was a significant potential barrier to taking PrEP upon release to the community. She remarked,

*For me*, *if I’m using*, *I won’t remember to take that*. *I’m pretty good about taking my medication when I am off drugs and stuff like that just because I have more common sense of myself*. *I have to take care of my kid*. *I guess if I just left it next to the kitchen when I’m about to eat or something*, *I’d remember to take it*. *20-year-old White Female- Sexual Risk*

Finally, some participants suggested that fear of side effects is an additional barrier that would prevent some individuals from wanting to take PrEP. One participant said:

*The fear*, *you know what I'm saying*, *because people don't know*. *They think when you're taking medicine that you're going to throw up or diarrhea or—it ain't nothing like that*. *It's just like taking a aspirin every day*. *39-year-old African American Male—MSM*

## Discussion

To our knowledge, this is the first study examining knowledge of and willingness to use HIV PrEP among people in a jail setting in the South. Our study found that healthcare is not often a priority post-release, knowledge of PrEP is low but willingness to take PrEP is high, perceived risk of HIV is low, and there are barriers to both disclosing sexual behaviors and assessing PrEP post-release. While many of these themes are consistent with current literature on post release priorities among people recently released from incarceration [6, 22] and literature on barriers to willingness to use and adhere to PrEP [17], our findings are contextually unique and therefore have important implications for future implementation of PrEP access either within jail settings or linkage to PrEP post release.

Previous research has shown that individuals face competing needs during reentry to the community and often healthcare is not a high priority. For instance, in a qualitative study that explored knowledge, interest, and anticipated barriers of PrEP uptake in the Rhode Island Department of Corrections, participants identified myriad barriers that affect PrEP uptake—including the hardships experienced during community re-entry [6]. Another qualitative study of women who were recently released from jail found that only 5 of 28 listed health care as their top priority [22]. Rather, the women included housing, employment, and family as reasons why health care was not on the top of their list.

Additionally, individuals within incarcerated settings may not perceive themselves to be at significant risk for HIV infection. In a recent survey study of incarcerated women who engaged in high-risk behaviors and who were eligible for PrEP, only 17% perceived themselves to be at risk for contracting HIV [23]. This mirrors our findings in that numerous participants in our study articulated a low perception of risk because they did not see themselves fitting into a specific risk group, such as IDU or MSM. Our data also suggest a relationship between perception of risk and stigma. For example, numerous participants articulated risk behaviors, such as IDU and same-sex behavior as sources of shame among their peers. These participants also alluded to feelings of broader social shame given that IDU and same-sex behaviors are often associated with considerable social stigmas. Again, this mirrors prior research among non-incarcerated populations [16, 18].

Disclosure of risk behaviors within correctional settings is a challenge, especially given the stigma associated with specific HIV related risk behaviors [6, 15]. Therefore, utilizing the CDC PrEP screening criteria might not be the optimal way to identify individuals who may benefit from PrEP. While some participants in our study found less trouble with disclosing drug-use behavior, many participants were less comfortable disclosing sexual behavior. This finding suggests the need to carefully consider how best to screen for PrEP eligibility within criminal justice settings. Importantly, screening for HIV risk during jail intake, as was done in our study, may be suboptimal given that individuals may not feel comfortable disclosing HIV related risk during a period when they may be experiencing the acute trauma of being incarcerated. One qualitative study of best practices for PrEP screening among MSM at the Rhode Island Department of Corrections, highlighted institutional distrust as a barrier to disclosing PrEP eligibility [15]. Therefore, prior recommendations support including medical staff and/or staff from external agencies when conducting HIV risk screening to assess eligibility for PrEP within correctional facilities [15].

With respect to barriers to taking PrEP, many study participants discussed fears relating to both medication side effects and to individuals in the community finding out that they were taking PrEP (and possibly confusing PrEP with antiretroviral medications used to treat HIV infection). This is not surprising given that more than half of our sample were individuals of color and prior literature has documented fear of PrEP side effects among individuals of color as a key barrier to uptake [17]. However, one important additional barrier endorsed by many participants in our study is participants’ perception that their lives post-release from jail may be too chaotic to be adherent to PrEP. Several participants expressed fears that they would relapse to drug use and/or return to the same environment that they were in before incarceration and therefore not be able to consistently take PrEP as indicated.

### Limitations

This study was conducted in a single county jail in the most urban area of very rural state, which may limit the generalizability of our results. All data collected were self-report, and some participants may have been unwilling to disclose sensitive information about themselves in this setting due to anticipated stigmas. Thus, our sample is further limited by including only those individuals who felt comfortable disclosing their HIV risk behaviors. Additionally, participants may not have felt comfortable self-reporting their preferred gender and/or sexual identity during the screening. Given that people reported low prior knowledge of PrEP, their responses to interview items likely represent a first impression of PrEP; their responses to interview items may have differed with greater prior knowledge and/or a more nuanced understanding of the requirements of this intervention. Another important potential limitation is the timing of the screening. As previously noted, we conducted our PrEP screening during the jail intake process, which is often a chaotic period with individuals dealing with the likely stress of having been arrested and being booked into the jail facility. Additionally, intake staff must collect a multitude of information in an expeditious manner in order to accommodate the volume of individuals they are seeing within relatively short periods of time. People being booked into the jail may also have significant reluctance to disclose any behavior they feel may impact their treatment from jail staff or other individuals detained within the jail. Therefore, we acknowledge that the intake period may not be an optimal time to screen for potentially sensitive information, such as HIV risk. Finally, we acknowledge the relatively small sample sizes within each risk category which prohibited us from stratifying our data by type of risk or demographic profile. Important and nuanced differences could exist for different risk profiles and future research should examine these more closely.

## Conclusion

This is the first study to specifically examine knowledge of and willingness to use HIV PrEP among people in a jail setting in the U.S. South. Results from our study suggest that often individuals do not (or are not able) to prioritize accessing healthcare upon community reentry due to competing needs such as housing and employment. We also found that among participants in our study, knowledge of PrEP was low but most individuals expressed willingness to take PrEP, despite their overall low perception of HIV risk. Finally, several important barriers to accessing PrEP and/or being retained in PrEP care upon reentry to the community include relapse to drug use, PrEP availability, the need to take PrEP daily to achieve maximum protection and stigma associated with taking PrEP (and/or disclosing risk behaviors which determine clinical indication for PrEP). Future PrEP and/or other HIV prevention interventions need to incorporate approaches to address the challenges individuals face during the period immediately post-release from CJ settings.
